# Synthesis of Two Decades of US EPA’s Ecosystem Services Research to Inform Environmental, Community, and Sustainability Decision Making

**DOI:** 10.3390/su13158249

**Published:** 2021-07-23

**Authors:** Matthew C. Harwell, Chloe A. Jackson

**Affiliations:** 1 US EPA, Pacific Ecological Systems Division, Newport, OR 97365, USA; 2 US EPA, Gulf Ecosystem Measurement and Modeling Division, Gulf Breeze, FL 32561, USA;

**Keywords:** ecosystem services, conceptual framework, final ecosystem goods and services, environmental decision making

## Abstract

A conceptual framework is helpful to understand what types of ecosystem services (ES) information is needed to support decision making. Principles of structured decision making are helpful for articulating how ES consideration can influence different elements in a given decision context resulting in changes to the environment, human health, and well-being. This article presents a holistic view of an ES framework, summarizing two decades of the US EPA’s ES research, including recent advances in *final* ES, those ES that provide benefits directly to people. Approximately 150 peer-reviewed publications, technical reports, and book chapters characterize a large ES research portfolio. In introducing framework elements and the suite of relevant US EPA research for each element, both challenges and opportunities are identified. Lessons from research to advance each of the final ES elements can be useful for identifying gaps and future science needs. Ultimately, the goal of this article is to help the reader develop an operational understanding of the final ES conceptual framework, an understanding of the state of science for a number of ES elements, and an introduction to some ES tools, models, and frameworks that may be of use in their case-study applications or decision-making contexts.

## 1. Introduction, Conceptual Framework

Over the past few decades, there has been an explosion in the literature on ecosystem services science. Notable milestones include: (1) a 1997 foundational text by Daily [1]; (2) a pair of central publications by Costanza et al. [2,3]; (3) the Millennium Ecosystem Assessment report [4]; and (4) the establishment of journals such as *Ecological Economics*, *International Journal of Biodiversity Science, Ecosystem Services & Management* (renamed *Ecosystems and People*), and *Ecosystem Services*. During this period, researchers at the US Environmental Protection Agency (US EPA) advanced ecosystem goods and services (collectively referred to as ecosystem services, or ES) science in multiple areas, including foundational research into metrics and indicators (including on those *final* ecosystem goods and services that directly benefit people), ecological production functions (EPFs), clear definitions to support standardized approaches for systematically classifying ES, and informing decision making based on advancing research on how human actions change the biophysical environment and ultimately affect the quality and quantity of benefits derived from nature. How people are affected by changes in availability of ES may influence decisions about whether to modify those actions. In addition to foundational research on frameworks, models, and tools, case-study applications provide valuable areas to test approaches and tools, help articulate development of transferable, organizational frameworks, and identify areas of future investment.

**Figure 1.**
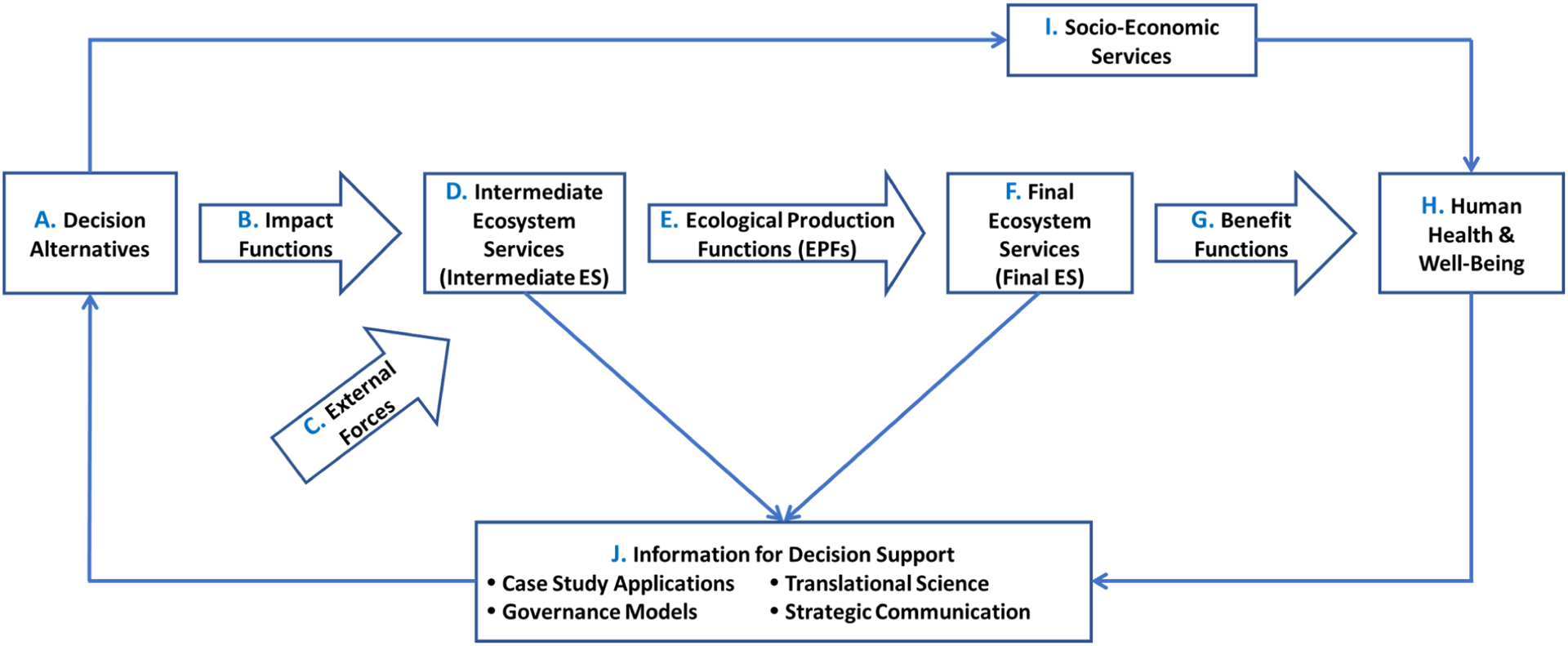
Conceptual framework for incorporating ecosystem services into decision making. Labels are referenced by Sections in this article. Source: modified from [8].

Along with investments in stakeholder engagement that integrates researchers, stakeholders (and prioritization), and decision makers, frameworks can create opportunities to explore value-added benefits of incorporating ES into a decision process [5]. A framework provides an opportunity to organize and communicate complex scientific information, creating the capacity to develop and apply transdisciplinary approaches to environmental decision making [6]. This synthesis paper organizes the extensive US EPA body of work within the context of a recent conceptual framework collaboratively developed with other US EPA researchers (Figure 1), which is helpful in understanding what types of ES information are needed to inform decision making. It is important to note that there are other recent examples of ES frameworks. For example, a cascade model [7] provides a good expression of some key components of a larger ES paradigm but does not include the larger context around ES as specific elements of decision support. The final ES conceptual model presented here, focusing on the importance of a beneficiary perspective, has helped expand beyond simple characterization of ES into categories of: provisioning services; regulating services; supporting services; and cultural services [4].

This paper is organized around scientific advancements associated with each element of the conceptual framework in Figure 1. In any decision-making context involving an environmental component, choosing among decision alternatives (**A**) can inform how those alternatives may influence the environment and lead to changes to human health and well-being (**H**). Each potential alternative involves actions, for which relationships between the actions and the impact of those actions (**B**) result in changes to the natural environment, including structure, function, and benefits provided by the environment (**D**). Here, it is also important to consider the influence of external forces (**C**), such as climatic factors, pollutants, and infrastructure, on changing ecosystem structure or function. In considering the status, condition, and ES provided by the environment (**D**), there is a subset of ES (**F**) that are *directly* connected to human health and well-being [9], referred to as *final* ecosystem goods and services (FEGS; also referred to as final ES). Ecological production functions (EPFs) are used to describe and measure changes in ecological structure or processes by which changes to the biophysical state of an ecosystem alter production of final ES (**E**) and are often estimated using ecological models. Final ES produce benefits that are measured by a human-mediated function (**G**) that conveys their value to human health and well-being (**H**). Importantly, these environmental benefits may be derived or influenced by social or economic services (**I**). Overall, these elements provide information to inform decisions (**J**) through decision-analysis tools, case-study examples, targeted communication, or governance-specific procedures. Further, closing an adaptive management loop, those lead to informing one or more decision alternatives. Overall, the suite of ES research described in this article touches upon each of the six areas identified in the Costanza et al. [3] twenty-years-after assessment of ES science: (1) the value of integrated ES (measuring, mapping, modeling, valuation) for decision support; (2) the use of ES to inform tradeoffs; (3) efforts on ES assessment and accounting; (4) alternative scenario analyses and modeling; (5) bundling rather than studying individual ES; and (6) a focus on ES issues related to scaling and transferability.

## 2. Literature Review Methodology

The US EPA peer-reviewed literature was identified through targeted searches, including Google Scholar, US EPA databases (e.g., Science Inventory), and communication with current US EPA ecosystem services science researchers. Peer-reviewed studies led by the US EPA involving a primary focus on ES science were included. Several US EPA research efforts where ES were tangentially mentioned were generally excluded; however, some areas, such as the extensive work on the US Human Well-being Index, where connections to services provided by the environment are incorporated are briefly discussed and the reader is sent to other resources to learn more (see Section 3.7). Examples of peer-reviewed, published studies on US EPA tools led by non-US EPA affiliated researchers were generally excluded, as in the case of many of the more than 70 published studies using the EnviroAtlas (see Section 3.5). Any US EPA peer-reviewed ES study not included is an unintended error of omission by the authors.

**Figure 2.**
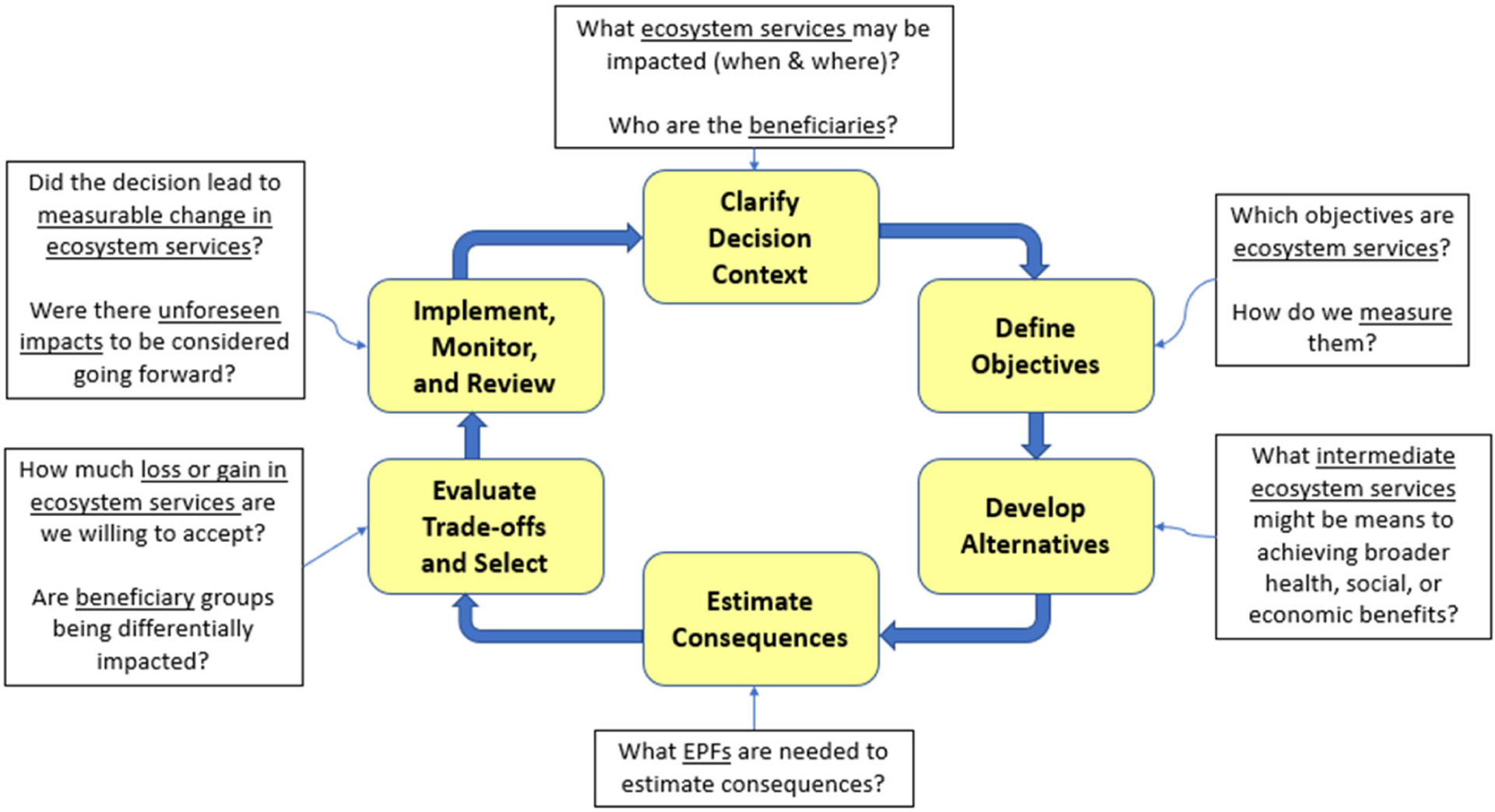
Example questions (white boxes) for mapping final ES concepts onto a structured decision-making framework (yellow boxes). EPF = ecological production function. Source: modified from [5].

A recent suite of in-depth case studies developed to operationalize the framework are presented at the end of this paper. Built from a range of experiences, a larger suite of ES assessment tools, models, and frameworks can be mapped onto elements of a structured decision making (SDM) framework [5] (Figure 2). This creates the opportunity to use the SDM framework as a translator (i.e., a “Rosetta Stone” approach) for mapping ES tools onto other decision processes (e.g., the National Environmental Policy Act process) without having to develop crosswalks between a suite of ES tools and each separate decision-making process. In contrast, a more targeted approach of introducing ES into a decision framework includes the development of Generic Ecosystem Service Assessment Endpoints for inclusion into the ecological risk assessment process [10–13]. Future US EPA research includes further developing [14] and applying [15] frameworks outlining connections between ES and contaminated site cleanup processes, including principles of greener cleanups [16], and other ecosystem considerations.

## 3. Results and Discussion—The US EPA’s Ecosystem Services Research Elements

### 3.1. Decision Alternatives (A) and Impact Functions (B)

Framing ES science in the context of informing decision making helps operationalize the application of ES concepts, ideally with focused efforts to connect ES directly to decision support tools and approaches. The cornerstone of the conceptual framework (Figure 1) is the role that decision alternatives (**A**) and impact functions (**B**) play in setting the overall decision context. There are many factors that can be used to inform decision alternatives, such as budget, time/space constraints, scientific/technical limitations, etc. Elements of SDM [17] and stakeholder engagement can lead to more robust decisions. For example, the US EPA has developed the FEGS Scoping Tool [18] to support inclusion of diverse stakeholders in discussions regarding development of decision alternatives in an ES context (see Section 3.5). The FEGS Scoping Tool uses the clearly defined categories of the National Ecosystem Services Classification System (NESCS) Plus [19] to help determine who may be affected by decision alternatives and what these beneficiaries value as some general (transition) language before digging into some of the more specific study findings. The other themes introduced in this section (identifying priorities, stakeholder prioritization criteria, stakeholder engagement, structuring tools and models around decision support, and introducing the value of case-study applications) are relevant to—and repeated throughout—the other elements of this framework (Figure 1) and this article.

Fulford et al. [20] used approaches linking stakeholder priorities to elements of human well-being. Both community stakeholder-engagement workshops and keyword analysis of community strategic-planning documents were used to examine the importance of identifying long-term stakeholder priorities as they inform decision-making goals. Keyword identification analyses can be a more objective way to use a community’s own efforts to define priorities; however, a strategic planning document’s structure may not follow a consistent format, and thus not result in a comprehensive analysis. There are challenges in achieving consistent and sustainable outcomes across multiple communities that differ in resource availability and management priorities, so Fulford et al. [21] used a keyword-based approach to look for common terminology in community objectives in strategic-planning documents. Comparing those to community demographics, location, and type, and looking for metrics of human well-being is useful for development of indices of community sustainability applicable to multiple communities with similar demographics, regional location, and type [21]. Yee et al. [22] further developed keyword analysis approaches by using a standardized classification system to develop a hierarchical list of final ES by analysis of planning documents for 28 National Estuary Programs and 29 National Estuarine Research Reserve Systems to be used for estuarine management planning efforts. Elements for understanding community decision-making contexts include consideration of four types of metrics [23]: community type [24]; human well-being index (HWBI; see Section 3.7); stakeholder priorities [25]; and availability of ES.

Advances in stakeholder prioritization in environmental decision making involve development of prioritization criteria based upon literature analysis in the environmental management, business, and public relations fields [25], and the incorporation of such criteria into ES tools such as the final ES prioritization tool, the FEGS Scoping Tool [18]. Orlando and Yee [26] provided another example highlighting the importance of using the process of stakeholder engagement to identify and prioritize stakeholder values and preferences and to identify tradeoffs among management alternatives.

Direct stakeholder engagement is efficient for identifying objectives; however, results may not be generalizable to the entire community or other communities. An alternative method of identifying environmental benefit preferences of a community is through analysis of social media photographs depicting preferences for active and passive ES-related activities as demonstrated in the St. Louis River and Milwaukee Estuary [27]. Littles et al. [28] looked at a different strategy drawing connections between people (users of the environment) and habitat classes (and subsequent ES) most relevant to them through a weight of evidence synthesis of the published literature.

Stakeholder workshop approaches can be used to look at how ES or human well-being elements are utilized in various processes (e.g., US EPA, state agency programs, local planning, and agency decision processes). This approach was used by Williams et al. [6] to advance community revitalization following sediment remediation and aquatic habitat restoration in a Great Lakes’ Area of Concern (AOC) community. Acknowledging the importance of stakeholder engagement, Gibble et al. [29] incorporated different types of stakeholder engagement at different organizational and temporal scales of multi-agency efforts to manage freshwater wetlands of the Everglades (Florida). Holifield and Williams [30] developed a methodology to use online Qualtrics survey and semi-structured interviews to identify appropriate geographic and governance scales to best apply ES lessons to inform decision making.

A variety of decision-support frameworks, tools, and approaches for developing and analyzing alternative scenarios have been developed and tested by the US EPA, including use in quantifying, assessing, and examining ES tradeoffs [5]. To operationalize frameworks in ES science, Russell et al. [31] integrated ecological, economic, and social values and information to address local metrics (importance to stakeholders, relative economic value, and availability of scientific information) in co-developing an ES program with stakeholders for Tampa Bay, Florida. In a different location, Sumner et al. [32] developed and tested an Alternative Futures Analysis framework approach for forecasting and quantifying (via Geographical Information Systems (GIS) analyses) the cumulative effect of management practices on future management ES in wetlands in the Great Salt Lake Ecosystem. Using the spatially distributed VELMA (Visualizing Ecosystem Land Management Assessments) ecohydrological tool for an application on green stormwater management practices such as Low Impact Development, Hoghooghi et al. [33] examined different watershed-scale decision alternatives through spatially explicit modeling of differently configured low impact development practices on watershed hydrology in the Pacific Northwest. In yet another GIS-based study, Angradi et al. [34] quantified changes in the spatial extent of ES provisioning in the St. Louis River Estuary, designated an AOC by the 1987 Great Lakes Water Quality Agreement, for management scenarios on sediment remediation and habitat restoration projects. Although originally developed for the Tampa Bay, Florida, watershed, Russell et al. [35] recognized the challenges of some GIS license costs to communities and used free and open-source GIS to develop a downloadable executable GIS-based EPA H_2_O tool used to quantify ES under different land-use scenarios applicable in multiple locations. Finally, Bayesian network analyses [36] can be applied as a technique to assess uncertainty in multi-dimensional management decisions, as demonstrated in coral reef management applications focusing on uncertainties related to stressors, coral reef condition, and related ES production [37,38].

### 3.2. External Forces

It is important to identify ways to explicitly incorporate the importance of external factors into environmental decision making. In our conceptual framework, external forces (**C**) are acknowledged as playing a concurrent role in influencing ecosystem structure or function. Examples include disturbance events, pollutants, changes in adjacent infrastructure, land use and management, and developing ecosystem-based management [39] strategies around multiple stressors and other climatic factors.

Bridging the gap between the challenge of having no ES assessment data to inform environmental decision making versus having in-depth ES evaluations, Myer and Johnston [40] described how to develop rapid ES mapping assessments to address stakeholder-driven needs for understanding resilience recovery and rebuilding on Long Island following Superstorm Sandy in 2012. Examining other external stressors, McKane et al. [41] described ES-relevant modeling of nutrients and contaminants entering estuarine ecosystems from terrestrial sources in a Puget Sound case study to inform land-use decisions. Examining a mixture of environmental and anthropogenic stressors, Weijerman et al. [42] analyzed ES and coral reef management tradeoffs resulting from a suite of local- and global-scale stressors.

Research on climatic factors affecting ES assessments include mapping changes in ES delivery resulting from alternative scenarios, with lessons learned from individual applications relevant to other decision contexts. Early examples include ES assessments related to optimal allocation and sustainable management of water and land resources in the Santa Cruz watershed ecosystem [43]. Recent research includes mapping-based analyses of ES-related property protection in the northern Gulf of Mexico under different habitat change and sea-level rise scenarios [44], and an analysis of metrics of ES and human well-being for alternative future scenarios examining different degrees of population growth and environmental resource protection in the same region [45]. Empirical modeling approaches also are used to examine alternative management scenarios in response to changing climate scenarios, including effects on water yield and rice production in the Lower Mekong Basin [46,47], and analysis of climate variables to identify robust predictors of vegetation associations for assessing bioclimatic shifts important for ES production assessments and ecosystem-based management [48].

### 3.3. Intermediate Ecosystem Goods and Services (Intermediate ES)

A systems approach to incorporating ES into environmental decision making involves understanding the larger suite of potential ES before focusing in on priority ES for a given decision context. Research on the concepts of final ES (see Section 3.4) starts with identifying and measuring intermediate ES (**D**) that contribute to the production of final ES which directly benefit people. For example, fish in the water that are caught by recreational fishers are a final ES, but an attribute such as water quality is an important intermediate ES that leads to the presence of the fish.

In a water quality intermediate ES example, nutrient retention by wetlands provides an important, intermediate step to final benefits such as clean water. Wetlands act as sinks for sediment, nutrients such as nitrogen (N) and phosphorous (P), and other pollutants [49–51]. Craft and Schubauer-Berigan [49] found that wetlands with hydrologic connectivity to other aquatic ecosystems enriched with nutrients retain more N and P than those with low anthropogenic nutrient inputs. Sierszen et al. [52] documented nutrient retention as an important ES provided by the Laurentian Great Lakes coastal wetlands. Jordan et al. [50] determined the amount of N wetlands remove worldwide is roughly 17% of anthropogenic N inputs and that wetland protection and restoration should be expanded to mitigate against excessive N loading. Kreiling et al. [53] examined the ability of restored wetlands in the Upper Mississippi River basin to remove sediment and nutrients before they flow into the river and fond that nutrient retention increases if more water is diverted into restored and natural marshes prior to reaching the river. These types of intermediate ES studies lead to better understanding of wetland management for improving the final service of water quality and their potential value in water quality trading programs.

The US EPA’s extensive ES research in the Great Lakes, the world’s largest collective repository of surface freshwater, has included many studies focusing on intermediate ES. For example, in the St. Louis River AOC, nine beneficial use impairments (BUI) were identified for removal, including excessive loading of sediment and nutrients to Lake Superior [54]. Bellinger et al. [54] analyzed 60 years of water quality data and determined that the concentration of nutrients and sediment had decreased enough to consider removing the Excessive Loading of Sediment and Nutrients BUI. Recognizing that a comprehensive inventory of ES provided by the Great Lakes did not exist, Steinman et al. [55] conducted a summit with 28 natural and social science experts, identifying several recommendations including developing an inventory of ES, addressing data gaps, and further developing tradeoff analysis strategies incorporating ES.

Another intermediate ES example is stormwater retention provided by the landscape, contributing to property protection. It is important to understand how changes in land cover alter stormwater regimes and this can only be done with up-to-date and accurate data. Reistetter and Russell [56] compared different methods of calculating an index of stormwater mitigation services provided by the landscape, developing recommendations on which datasets and methods to use. In a different example, the presence of coral reefs provides a buffer from storms and waves, contributing to property protection [26]. Orlando and Yee [26] identified how sediment runoff impacts the ES provided by coral reefs finding that higher sediment delivery decreases production of most ES (e.g., ecosystem integrity, bioprospecting discovery, recreational activities, fisheries production) but increases the service of property protection. Their results highlight the importance of using the process of stakeholder engagement to identify and prioritize stakeholder values and preferences and to identify tradeoffs among management alternatives.

Research has focused on moving beyond mapping the presence of—or applying a dollar value to—a given ES and into developing more targeted information that can be used to inform decision support. Early US EPA research focused on measuring the effectiveness of environmental programs in terms of ES through a Relative Valuation of Multiple Ecosystem Services Index, which assigns a value to ES (both intermediate and final) and expresses the output in either relative units or in dollar value [57]. More recent US EPA research has focused on ES and decision support tools, such as the Rapid Benefit Indicators (RBI) approach, a systematic approach to compile non-monetary benefits indicators [58,59]. These decision support tools are described below in Section 3.6.

### 3.4. Ecological Production Functions (EPFs)

There is a need to turn ES into something tangible for bringing into a decision process. One of those important efforts involves ecological production functions (**E**), useable expressions that characterize (model) ecosystem processes by which final ES are generated [60]. Recognizing that final ES themselves do not always need to be quantitative to inform decision making, Bruins et al. [60] identified core attributes for EPFs in the context of advancing ES science to best support decision making:

- Estimate indicators of final ES;
- Quantify ES outcomes;
- Respond to ecosystem condition;
- Respond to stressor levels or potential management scenarios;
- Appropriately reflect ecological complexity;
- Rely on data with broad coverage;
- Are shown to perform well;
- Are practical to use; and
- Are open and transparent ([60], p. 54).

Two EPA contributions to EPF science include an online, searchable database (EcoService Models Library; ESML) containing 50 individual descriptors for finding, examining, and comparing ecological models in the published literature [61], and a new approach that characterizes a given model’s “application niche” [62], both aimed at informing transferability of models/equations to other ecosystems/contexts and generalizability. The ESML functions as a metadata repository on a suite of EPFs available in the literature [61]. Moon et al. [62] developed an analytical technique to characterize a given model’s application niche through synthesizing information from databases, past studies, and models in original and novel applications to develop performance curves characterizing whether a given model is appropriate or not for a different context.

Fulford et al. [63] identified four issues associated with model-based assessments of ES, including: (1) choice of model being well-suited to issue and location (i.e., the same issues identified by [61,62]); (2) appropriate level of complexity necessary to address a management problem; (3) translation of model output into the language of policy; and (4) proper engagement with decision makers so they accept and use model-based information. The US EPA’s EPF science efforts have primarily focused on advancing core EPF attributes [60,62] while keeping in mind principles/issues associated with modeling ES [63] across a suite of marine (e.g., corals, coastal/estuarine), freshwater (lakes, rivers/streams, wetlands), and terrestrial (agroecosystems, forests) environment types. While most modeling has focused on EPFs related to informing valuation of ES for direct use purposes (e.g., forest harvests), additional research has included EPF work related to non-use, existence values, and intrinsic benefits from nature ascribed to their presence. For example, Marcot et al. [64] modeled habitat structure ultimately related to existence values for the Northern Spotted Owl, and Fordham et al. [65] connected an individual-based model to a niche-population model to examine turtle species in tropical savannahs. In a different type of ES modeling context examining how the human environment has impacted ES, an ES-Life Cycle Impact Assessment conceptual framework was developed [66] and case-tested [67] by modeling environmental cause–effect chains (on resource use or pollutant emissions) into potential environmental impacts of supply chains and their products.

#### 3.4.1. EPFs in Marine Systems

Research on EPFs in marine ecosystems includes corals, coastal/estuarine habitats, and fisheries contexts. Principe et al. [68] presented a literature survey on identification and characterization of coral reef ES that captured information on measuring and valuing coral reef services as indicators useful for management and sustainable-use planning. Existing ES quantification methods are summarized related to ecological integrity, shoreline protection, recreational opportunities, and fisheries production [69], and potential for natural products discovery from reefs [70].

In coastal and estuarine systems, recent ES science has focused on using a habitat perspective as “a practical method for developing models (or, ecological production functions, EPFs) to describe the spatial distribution of ecosystem services” [71]. Jordan et al. [72] argued for using a habitat-focused, rather than just a final ES–focused, perspective for considering ES in coastal, estuarine, and fisheries contexts by describing challenges based on mismatches between the measurement of EPFs at small spatial and short temporal scales versus delivery of coastal, estuarine, and fisheries ES over extensive scales of space and time. In a location-specific study, Lewis et al. [73] summarized ES and environmental information for the Pensacola Bay, Florida, system to develop 20 recommendations for research modeling, habitat restoration, and system monitoring to improve the condition of the system. Ayvazian et al. [74] presented an oyster habitat restoration project example focused, in part, on improving intermediate ES of fish and invertebrate refuge and foraging habitat. In another oyster example, Bricker et al. [75] examined the economic value for aquaculture-based removal of N, in part to inform management and tradeoff decisions related to aquaculture and reef restoration.

The domain of ecosystem-based models relevant to characterizing ES in coastal and estuarine habitats, including both challenges in ES modeling and the integration of ES models into coastal management decision-making processes, is presented by Lewis et al. [76]. They argued for co-development of modeling strategies among scientists, resource managers, and relevant stakeholders [76]. Those strategies can be enhanced by surveying local users to identify coastal habitat–related user/habitat dependencies to help prioritize EPF characterization and local ES valuation to support decision making [28]. Finally, Fulford et al. [77] presented a logic argument for the connection between ES and the preservation of ecosystem function, applying the fisheries science concept of functional equivalency, where desired ES functions are managed to preserve (e.g., a sustainable fishery harvest) while allowing for general change in the ecosystem, to inform environmental decision making in a broader context outside of marine/coastal ecosystems.

#### 3.4.2. EPFs in Freshwater Systems

Research on EPFs has been advanced in freshwater lakes, rivers/streams, and wetlands. Integrating quantitative data on multiple species and habitat components provides a multimetric context for EPF analyses. In a study characterizing Great Lakes fisheries, Trebitz and Hoffman [78] analyzed data on ecosystem components and information on commercial and recreational harvests to inform fishery management. To connect riverine ecosystem conditions and resultant ES functions, Weber and Ringold [79,80] used a beneficiary survey approach (interview and focus group data) to identify specific, publicly relevant river features to organize the development of monitoring design and measurement of aquatic EPFs for river management decision making.

Examples of US EPA EPF research in wetland settings include both large-scale, broad assessments [81] as well as small-scale, detailed experimental efforts [82,83]. As an example of how to connect potential loss of ES associated with loss of habitat to examine management issues such as wetland mitigation and natural resource damage assessments, Engle [81] examined ES across Gulf of Mexico coastal wetlands related to essential habitat for juvenile shrimp production, storm surge protection, water quality improvements by N removal, and carbon sequestration. Wetland EPFs can be used to connect productivity and long-term carbon sequestration rates in the context of changing environments. Herbert et al. [82] used this approach to examine the effects of chronic saltwater intrusion, and Li et al. [83] used this approach to examine projections of human population changes related to increases in temperatures, river discharge (via variable precipitation changes), and increasing freshwater withdrawal. In this pair of studies, Herbert et al. [82] and Li et al. [83] examined fine-scale experimental measurements of primary production in tidal fresh marshes dominated by the C3 grass *Zizaniopsis miliacea*.

Alternative ways to examine ES and EPFs in aquatic systems have focused on a watershed perspective at the large spatial scale of catchments, and at smaller, sub-watershed scales. Hill et al. [84] followed ES from EPFs through to a benefits assessment using economic conversion factors focusing on ES related to biodiversity, climate regulation, recreation, timber production, crop production, water supply, and water purification. Connecting ES and EPFs at this scale can be used for alternative land-use scenarios. In an example of an economic assessment, Teague et al. [85] used a watershed-scale approach to examine a suite of ES production in the Tampa Bay, Florida, region looking at alternative future land-use scenarios to conduct benefit assessments of alternative scenarios. Watershed-scale assessments can be used to examine ES that are traditionally difficult to relate in terms of monetary value (e.g., carbon storage and sequestration; water recharge; habitat support for biodiversity; and N and other “chemicals of concern” removal) as demonstrated by an assessment of water-level influence on both biogeochemical cycles and water supply recharge within the major river watershed in Tampa [86]. At an even smaller, neighborhood scale, Russell et al. [87] looked at functional, technical, and data considerations of GIS to map EPFs for air pollution removal, shading, carbon sequestration, N removal, walkability, access to greenspace, aesthetics of residential areas, and water-feature viewscapes to conduct benefits assessments.

#### 3.4.3. EPFs in Terrestrial Systems

In terrestrial EPFs, US EPA research has been done in agroecosystems and forest systems. Agroecosystem research has focused on system-level, integrated modeling of N fate and transport processes in the Mississippi River Basin [88–90]. A multimedia modeling approach can be used to assess impacts of fertilization, meteorology, and atmospheric N deposition on water quality and the export of N to the Gulf of Mexico. Additional research has been directed towards informing land use and land management of agrosystems [89] and efforts to reduce the areal extent of the Gulf of Mexico hypoxic zone [88].

In forest ecosystems, EPF research has focused on modeling landscape changes resulting from different management/climate scenarios [91,92]. Using VELMA to link hydrological and biogeochemical processes within watersheds, Abdelnour et al. [91] examined effects of forest management under different climatic conditions on the condition and functions of forest and adjacent streams. In a later study, Abdelnour et al. [92] examined changes in terrestrial, aquatic, and atmospheric carbon and N dynamics before and after timber harvesting in the western Oregon Cascade Range.

Overall, EPF advances solve challenges of including ES in decision making resulting from limited understanding of linkages among ecosystem components and processes that ultimately deliver ES. In addition to further incorporation of ES elements into ecological models, future EPF research should focus on developing science related to ES trade-off assessments, informed, in part, by identification and incorporation of added ecological complexity into EPFs [60].

### 3.5. Final Ecosystem Goods and Services (Final ES)

Much US EPA research focuses on final ES (**F**) because final ES are generally easier to measure and communicate to stakeholders, avoid double counting issues, and can be directly connected to people’s values and their well-being [8]. Using final ES to value the benefits provided by the environment helps ensure collected data are more useful for society. Weber and Ringold [93] used both quantitative and qualitative techniques to identify ecological benefits for rivers and streams, and Ringold et al. [94] presented a six-step framework to identify final ES metrics of a stream.

**Figure 3.**
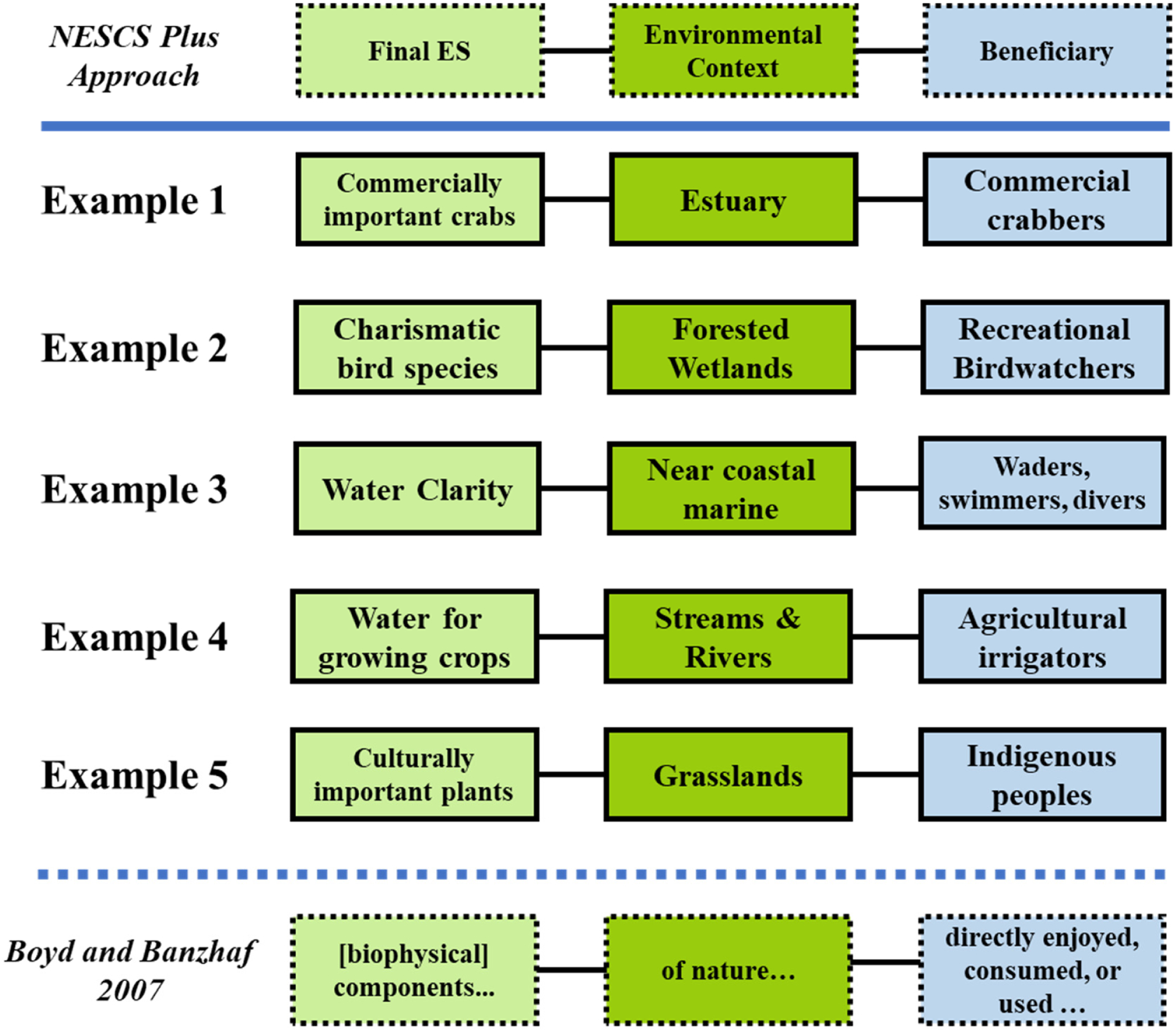
Examples of final ES in relation to the NESCS Plus structure [19]. For comparison, Boyd and Banzhaf [9] terminology is shown below the dashed line. The colors match those from Table 1. Source: modified from [8].

Using a beneficiary approach to classify ES helps determine who benefits and in what ways, and improves communication to stakeholders and policy makers. DeWitt et al. [8] (Figure 3) provides examples for integrating final ES into ecosystem-based management and other decision frameworks. To help address the challenge of identifying who those beneficiaries are in a community or at a particular site, Sharpe et al. [18] introduced the FEGS Scoping Tool to help decision makers in the early planning stages of a project to prioritize: (1) stakeholders [25]; (2) beneficiaries; and (3) attributes that the beneficiaries care most about.

**Figure 4.**
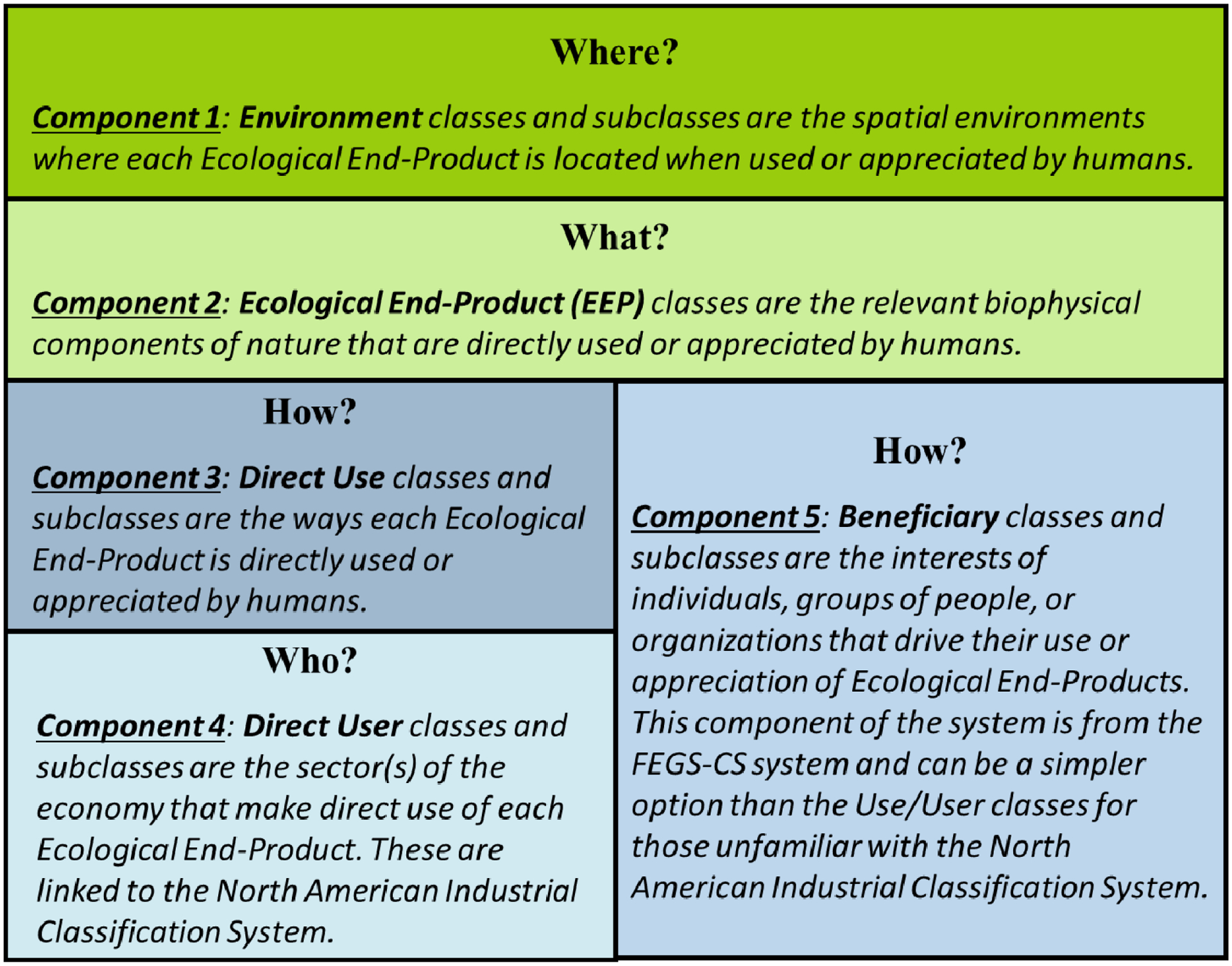
Core components of NESCS Plus and the focal questions (where, what, how, and who) used to systematically generate systematic final ES. The colors match those from Figure 3. Source: [19].

Classification systems provide standardizations of final ES terms, which lead to many benefits to practitioners that include: defining ecosystems more precisely; ensuring easier transferability of knowledge among scientists and studies; improved communication; and avoiding the need to recreate systems used to define and classify ES [95,96]. The US EPA’s FEGS Classification System (FEGS-CS) described and accounted for the final ES provided by a certain environment type for a specific beneficiary [97]. The US EPA’s National Ecosystem Services Classification System (NESCS) presented a framework for classifying ES to aid in analyzing the impacts of policy-induced changes to ecosystems on human health [98]. The US EPA recently combined both the FEGS-CS and NESCS classification systems into one classification system, called NESCS Plus, by combining the desirable features of both FEGS-CS and NESCS to give NESCS Plus the flexibility in offering two ways to classify the human dimensions [19]. The core components of the NESCS Plus Classification System (where, what, how, and who) are shown in Figure 4, and a detailed explanation on how to walk through the where, what, how, and who questions are provided in [19]. Newcomer-Johnson et al. [19] also provides detailed definitions of classes and sub-classes of environment, ecological end-products, use (i.e., distinct ways in which end-products can be directly used or appreciated by humans), user (i.e., the economic sectors through which people directly use or appreciate end-products), and beneficiary classes.

Distinguishing between intermediate ES and final ES can be tricky, so Johnston and Russell [99] presented operational guidelines on what to count when evaluating final ES. A 2016 methodology utilizing expert workshops [100] proposed how to develop metrics and indicators of final ES; this work was further developed by [101] in presenting a sequence of steps for proposing final ES metrics (Table 1).

Related to metrics and indicators, the EnviroAtlas is a US EPA online data and tool repository [102] containing more than 400 geospatial layers coded with seven categories of (intermediate and final) ES: food, fuel, and materials; clean air; recreation, culture, and aesthetics; natural hazard mitigation; climate stabilization; clean and plentiful water; and biodiversity and conservation [103]. At present, the EnviroAtlas has been used in more than 70 peer-reviewed publications; the reader is directed to US EPA [103] to learn more. Tashie and Ringold [104] used the final ES framework to assess whether systemic biases were introduced to ES assessments when only using land use and land cover data (from EnviroAtlas) and found major gaps in identification of beneficiaries. The results are compiled in a searchable and publicly available database useful for navigating EnviroAtlas data [105].

The US EPA conducts work on linking changes in stressors to changes in final ES. A STEPS (Stressor-Ecological Production function-final ecosystem Services) Framework allows policy makers, regulators, and land managers to determine the tradeoffs between pollution and protection for various ecosystem components. The STEPS Framework linking changes in biological indicators of a stressor to final ES was tested (using the FEGS-CS) to describe final ES and exceedance of critical loads of air pollution as a stressor [105]. O’Dea et al. [106] used the STEPS Framework and FEGS-CS in an exercise aimed to link aquatic acidification to the ES and beneficiaries impacted by this pollution. In another STEPS framework application, Rhodes et al. [107] examined linkages in aquatic eutrophication to final ES through linkages to phytoplankton community shifts in response to changing nutrients. In the ESML described earlier, the Variable Relationship Diagram is a systematic, visual approach for displaying connections between predictor variables within models to output endpoints allowing the user to quickly visualize relationships among predictor variables and how those variables contribute to processes contained within a model [61]. This advancement in ecological modeling improves communication of important functions and relationships to better help analysts and modelers see ways to connect models together to produce new and more powerful predictive tools.

### 3.6. Benefit Functions

A fundamental area of ES science relevant for providing decision support is the body of work focused on capturing the specific benefits of ES themselves for a given decision context. The change in a system brings about a change in ES, which we can predict using EPFs, and those ES provide benefits, which we can estimate using benefit functions (**G**). The science of measuring, understanding, and utilizing information on the benefits of ES in environmental decision making has multiple dimensions [108]. The two core pillars in ES benefits characterization are: measurement and mapping; and valuation. Research on measurement and characterization of benefits includes focus on metrics and indicators [109] and on mapping [110]. Research on valuation includes focus on characterizing the economics of ES [111–113].

**Figure 5.**
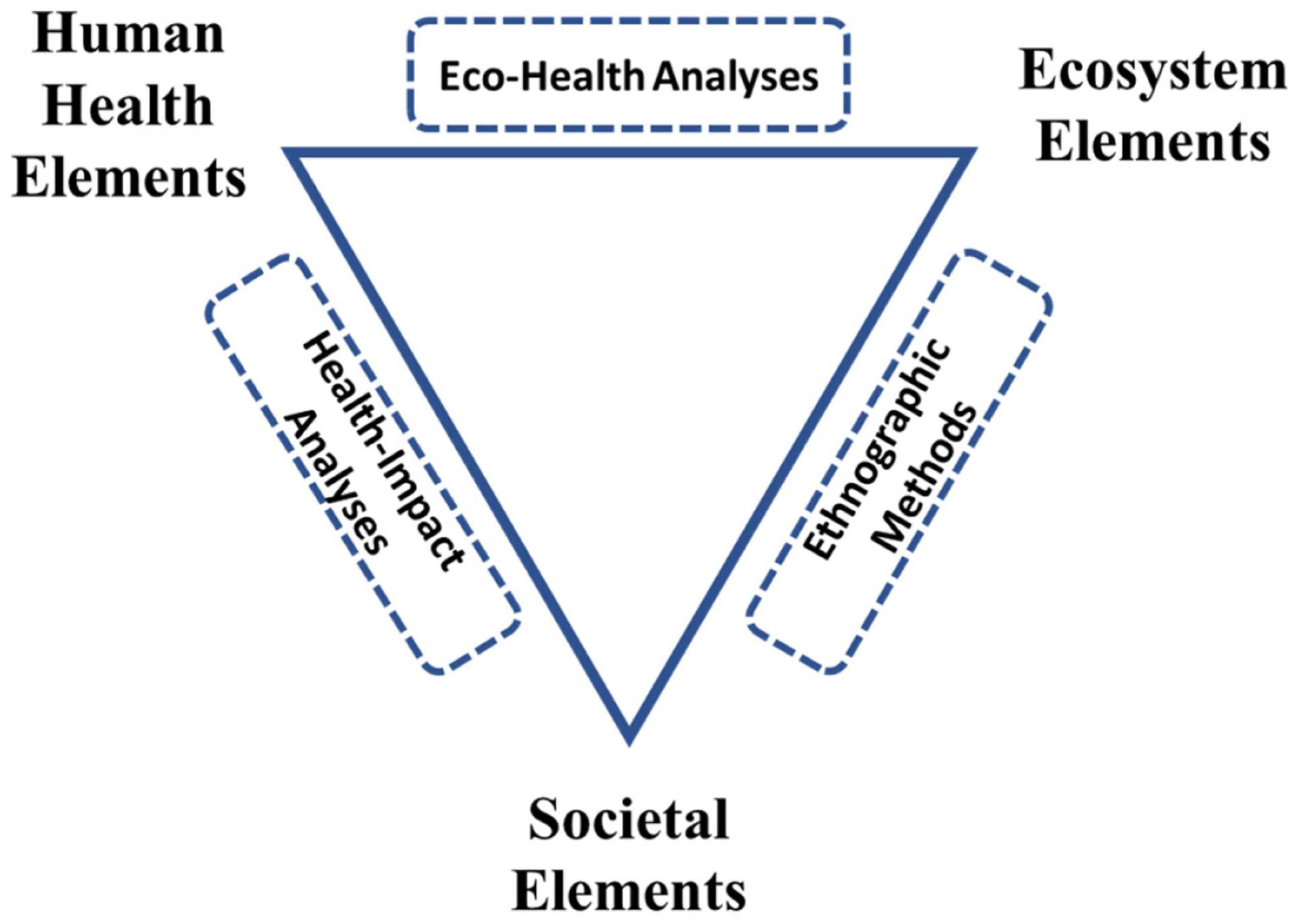
Examining the benefits from ES to human health and well-being among ecosystem, societal, and human health elements. Source: modified from [114].

In the field of non-monetary benefits of ES, Johnston et al. [114] characterized three types of approaches (Figure 5) looking at the intersections between: (1) ecosystems and human health elements (referred to as “eco-health” analyses) [114–117] described in Section 3.7; (2) societal and human health elements (via Health Impact Assessments; HIA) [114,118,119]; and (3) ecosystem and societal elements (via ethnographic methods such as the neighborhood scale analyses [6]). The US EPA’s HIA and ES benefits research are compiled in [118]. As these studies are interdisciplinary in nature, involving practitioners of social, economic, human health, and ecological sciences, readers need to be aware that differing lexicons and perspectives may impede communication [114]. The different components of “benefits” are not uniquely distinct research areas; rather, there are significant overlaps among them in both development and application.

Metrics and indicators of ecosystem benefits are important to connect the value of ecosystems to human beneficiaries. The term “linking indicators” refers to those biophysical indicators that inform interpretation of ecological conditions and change from a societal perspective in that they measure biophysical things directly affecting people’s welfare [109]. Indicator-based methods can be used where direct economic valuation is either too complex to undertake, or otherwise inadequate to provide a complete picture [59]. In an application on recreation-focused benefits of ES, Angradi et al. [116] examined how to develop metrics of benefits of good water quality for lakes, comparing subjective visual assessments quality (i.e., those that inform an individual’s perspective on the value of a lake for recreational purposes) to environmental monitoring data of water clarity, including Secchi Disk depth, turbidity, and water-column chlorophyll-a concentration data. Lomnicky et al. [120] developed a recreational fishery index as a way to connect ecological condition in rivers and streams and sport fishery ES. Benefits mapping of ES often includes combining traditional mapping with economic translations as demonstrated by Russell and Greening [110] combining biophysical mapping data for N removal and carbon sequestration with economic converters for estimating and mapping benefits associated with water treatment and offsetting carbon emissions.

Not surprisingly, evaluation and assessment of ES benefits can provide direct information to support decision making. In addition to other decision support examples in this section, the RBI approach involves five steps of presenting elements of benefits assessments to stakeholders in examining different management alternatives for a decision: (1) describe the decision context; (2) select ES and describe benefits; (3) compile benefit indicators; (4) summarize the indicators; and (5) use the results in decision making [58]. One example application of the RBI approach conducted in the urbanizing Woonasquatucket River Watershed (Rhode Island) demonstrated how to capture social benefits from small urban sites with relatively low ecological function [121]. In another RBI application, Bousquin and Kristen [122] assembled a national dataset of spatial indicators to allow communities to more quickly screen restoration and conservation projects based on potential flood reduction benefits, with a green infrastructure-focused application in Harris County, Texas.

#### 3.6.1. Economic Valuation

Applications of economics valuations include, but are not limited to, use in decisions related to policy development, alternative selection or tradeoff analyses, market-focused applications, and use in communicating the importance of nature to people. Given that most environmental resources are provided freely by nature, they typically are not paid for by people; however, environmental decision making often involves balancing components of the environment with other things valued by people. As a result, economic applications of ES are sometimes needed to inform decision making. An overview of terminology, such as the four types of non-market values (direct-use values; indirect use values; option values; and non-use values) is provided in [112]. They describe a range of economic methods that may be employed for analyses, including cost-effectiveness analysis, economic contribution analysis, economic impact analysis, or economic benefits analysis. In a survey of natural resource managers and their use of economic valuation surveys, ref. [123] three areas of interest were identified: (1) economic contributions and/or environmental impacts to the local economy; (2) values of the environment that the public is willing to pay for/give up for a management decision; and (3) the total, overall value of assets provided by an ecosystem. There is a general need to develop benefit estimates as part of regulation development; Weber [111] presented approaches to non-market ecological valuations in the context of regulatory support for the Clean Air Act and Clean Water Act using examples including valuation of secondary National Ambient Air Quality Standards. In the context of markets, Heberling et al. [124] presented a water quality trading model to illustrate how social benefits and costs can be explicitly considered to explore elements of a water quality trading program for wetlands and meeting water quality goals.

In the related field of natural capital accounting, accounts are developed to assess contribution of ecosystems to the economy whereby an ES can be considered as representing a type of “transaction” between humans and the natural capital of the environment [113]. Warnell et al. [125] developed an application of natural capital accounting by compiling ecosystem extent, condition, and ES supply and use accounts across a suite of ES for a 10-state region in the southeast US, illustrating how information from ecosystem accounts can contribute to policy and decision making.

From a communications perspective, economics valuation was presented as part of important stakeholder engagement in the Tampa Bay watershed in their restoration efforts by [110]. In another example on economic perspectives in communicating the benefits of ES, Weber et al. [126] examined economic functions related to recreational access to nature for the Sonoran Desert featuring a perennial stream, looking at both time-varying environmental factors related to quality of recreation and sociodemographic variables.

#### 3.6.2. Health Impact Assessments

Health Impact Assessments are “a means of assessing the health impacts of policies, plans, and projects in diverse economic sectors using quantitative, qualitative, and participatory techniques” [127]. The HIA process involves cooperative, transdisciplinary efforts that involve stakeholders and decision makers in the process of identifying potential impacts of concern to a community for a given decision. The HIA process was successfully used in a Suffolk County, NY, case application to identify ES and health impacts of interest and concern to the community [114]. Williams and Hoffman [119] used HIA in a Great Lakes AOC habitat restoration project to identify and connect potentially impacted ES as a result of the environmental cleanup project to endpoints such as swimmable water or edible fish, which could be connected to health benefits. Although HIAs are not considered an ES tool per se, these examples show that HIA can be used as an organization and communication tool to aid in community-scale ES assessments.

#### 3.6.3. Ethnographic Methods

Johnston et al. [114] presented an overview of the suite of US EPA’s conceptual modeling approaches developed for ES benefits analyses. The development and application of ethnographic methods—a suite of qualitative and quantitative social science methods used to observe and learn interactions in and around a particular setting or environment—can be used to translate ES information into information used in on-the-ground decisions. An ethnographic approach to studying the benefits of ES enables the identification and characterization of the different values placed on an ecosystem and its services [114]. These methods have been used for conceptual modeling of social-ecological systems, stakeholder engagement, and communication. For example, Williams et al. [6] used ethnographic methods to develop conceptual models demonstrating how to improve transparency and facilitate community-scale conversations and characterize barriers and constraints involving decisions and ES.

### 3.7. Human Health and Well-Being and Socio-Economic Services

To operationalize ES for decision support, connections need to be made between the potential benefits of ES to people and changes in human health and well-being. The US EPA has advanced research on developing ES connections with human health and well-being endpoints (**H**), including incorporating influences by socio-economic services (**I**). Beyond individual benefit functions and assessments, research has advanced on relationships to human well-being (including applications to community decision making), eco-health connections (including advancing work in HIA), and work on targeted human-health endpoints (e.g., impacts from extreme weather, flood hazard mitigation, gastrointestinal illness, and vector-borne diseases). In environmental, community, resilience, or sustainability decision making, it is important to recognize that elements beyond ES are also influential in a given decision making context. Although this synthesis is focused on ES, consideration of socio-economic services is relevant. There are a number of examples of relevant overlaps between research on ES and socio-economic services, including connections to developing ES benefit functions (Section 3.6), US EPA work on the Human Well-being Index (below), and example on-the-ground applications (e.g., see Section 4.2).

The HWBI, developed around the three sustainability pillars (social, economic, environmental), identifies domains of human well-being and relationships between social, economic, and environmental services to economic, environmental, and societal well-being [128]. Smith et al. [128] outlined the approach of connecting the domains of well-being to ES, including the need to evaluate ecosystems based on both the sense of security and opportunities for interaction with nature that ecosystems provide to people. The reader is directed to learn more on the HWBI, including details on indicators and methods [129], scaling [130], and adaptation/application to targeted populations such as Native American populations [131] and geographic regions (e.g., Puerto Rico) [132]. In a different HWBI application, Fulford et al. [24] looked at what a given community values (including benefits of nature) and connected those values to elements of human well-being. Fulford et al. [24] and Fulford et al. [23] developed a community classification system that groups like communities based on socio-demographic, economic dependence, and ecological characterization data and, along with HWBI information, developed an approach to answer the question, “Does community type provide a local reference point for measuring change?” Yee et al. [133] examined novel techniques for downscaling data (from municipal to census-tract scales) in the San Juan Bay estuary watershed (Puerto Rico) to examine how small spatial scale information on community well-being can inform environmental management impact assessments including on environmental justice inequalities among neighborhoods. In a final example, Yee et al. [45] examined effects of land-use change scenarios on human well-being through changes in ES production and delivery for the Pensacola Bay watershed, Florida.

There has been a significant increase in the overall science literature evaluating the eco-health connections between ES and human health and well-being endpoints. Recognizing that this field is dominated by observational research, one area that the US EPA has focused on is synthesis and integration of the literature. Bolgrien et al. [134] and Johnston [118] presented descriptions and applications of eco-health tools, such as the Eco-Health Relationship Browser and the EnviroAtlas. The Eco-Health Relationship Browser is a user-driven, online navigation tool that explores more than a decade of studies on the buffering and health-promotional aspects of ES [115]. Addressing the dearth of primary studies establishing causal associations between ES and human health, de Jesus Crespo and Fulford [117] developed a causal criteria analysis of the eco-health literature focusing on the context of green spaces providing buffering services that may influence diseases such as cardiovascular disease, heat morbidities, and respiratory illness. Other examples of research establishing connections between ES related to green space and human health endpoints includes identifying an inverse relationships between forest and near-road tree canopy and childhood autism rates [135], an inverse relationship between greenway density and percent forests and sudden unexpected deaths [136], and the protective effects of greenspace on Alzheimer’s disease [137]. Additional published eco-health studies include research establishing positive relationships between neighborhood greenery and reduced odds of being overweight or obese [138,139], street tree cover and metrics of active transportation lifestyles [140], vegetated land cover near residences and biomarkers of neuroendocrine, immune, metabolic and cardiovascular system functioning [141,142], and small but significant improvements in women’s self-reported general health and green cover, likely subject to the type of natural environment and urbanicity [143]. Summers and Vivian [144] synthesize the literature on ecotherapy, the ability of interaction with nature to enhance recovery from physical and mental illness.

Examples of targeted research related to human-health endpoints include work on vector-born disease and the potential for ES mitigation of disease. In a study on flood protection ES in Puerto Rico, De Jesus Crespo et al. [145] looked at benefits along with potential socio-economic confounding variables to examine human health concerns around gastrointestinal illness, ultimately informing management decisions relating to flood protection decisions. In a study on Dengue fever, de Jesús Crespo et al. [146] determined that after controlling for population density and other socio-economic aspects, higher percent wetland cover—and its resulting heat hazard mitigation benefit—reduced disease occurrence. Through mapping spatial distributions and using a Bayesian modeling approach, Myer et al. [147] found that although septic systems were associated with an increase in West Nile Virus, land cover classified as open water and woody wetlands was negatively associated with West Nile Virus incidence in Suffolk County, NY, suggesting that wetland cover has a mitigating ES benefit on infection in mosquitoes. Myer and Johnston [148] extended this work, applying an analytical technique to surveillance data to identify locally important predictors and ultimately improve West Nile Virus incidence models for use at the county and community scale. Additional applications of Bayesian modeling approaches for eco-health research include examining seasonal weather variation, vegetation height, human population, and land cover to examine mosquito-borne disease vectors in southern Texas [149]. In Puerto Rico, the ES benefits of wetlands is studied in relation to disease vectors in mosquito-borne illnesses, such as the Zika virus, ultimately informing management decisions on flooding reduction and water quality improvement in support of traditional methods to control spread of vector-borne diseases [150,151].

### 3.8. Information for Decision Support

Not surprisingly, elements throughout this manuscript have direct connections to decision support (**J**) and much of the content, including Section 3.1 are relevant here. Regardless of the process, tools, or frameworks used, focusing on the decision context provides a valuable anchor for exploring decision alternatives [5], and may create opportunities not elsewise identified. For example, watershed-focused organizations working on green infrastructure opportunities, and the resulting ES benefits, in Cincinnati’s Mill Creek were able to qualify for grant funding because they created strategic plans that informed decision alternatives, helping identify a suite of process opportunities [152]. Other areas of decision support not mentioned previously include applications and relevance of final ES to policy applications, and the importance of using/managing language for communication.

Evaluating environmental policies requires estimating impacts of policy-induced changes on ecological and human systems. Intermediate ES and final ES were examined as part of an analysis on impacts of secondary National Ambient Air Quality Standards for N oxides and sulfur oxides [153]. The concepts of final ES are very useful for decision support and can help support policy analysis by drawing important linkages between ecological and economic models. Final ES can also help inform the design of valuation studies that are more conducive to benefit transfers [154]. Sinha et al. [154] compared a set of existing valuation studies to the final ES approach to illustrate ways in which using final ES metrics could provide economists a useful starting point for considering how the commodity could be defined and specified in a valuation study. This approach is useful for determining the extent of uncertainty associated with the analysis and provides transparent documentation that can be informative for policy makers [154]. Bolgrien et al. [134] highlighted data and tools (e.g., EnviroAtlas, Eco-Health Relationship Browser) that the US EPA is providing to federal, state, tribal, and local partners to incorporate ES concepts into public policy decisions and environmental problem solving. These translational tools make scientific information and approaches more practical, relevant, and accessible so that a wider and more diverse range of stakeholders can make more informed decisions when addressing problems such as land use and social policy [134].

Finally, successful use of ES research for decision support depends on both standard terms and definitions and targeted efforts at communication. Munns et al. [155] provided a standard lexicon for ES as an important step towards standardizing language. Harwell et al. [156] developed a generalizable framework and organizational structure for strategic science communication, including guidelines for scientists, stakeholders, and decision makers, using an example of communicating ES benefits. Importantly, Harwell et al. [156] encouraged researchers to develop and implement a communication strategy at the beginning of a project instead of waiting until research is complete.

## 4. Results and Discussion—Operationalizing the US EPA’s Approach

Developing case-study applications across a range of ecosystems and environmental management decision contexts allows for identifying best practices and opportunities for transferability of tools and approaches [5]. The US EPA operationalized concepts developed through research and community engagement through case studies examining one or more individual elements of the final ES conceptual model (Figure 1). One case study approach used a place-based study strategy, allowing a holistic approach to operationalize scientific information for decision making through the integration of science with the social, economic, and environmental characteristics of a place [20]. Fulford et al. [20] evaluated the final ES conceptual model (Figure 1) at the community level in the context of existing and previous place-based studies by surveying place-based study teams throughout the US and Puerto Rico to ask how their research has (or not) applied elements of the final ES conceptual model for decision support.

At several study sites, the US EPA did extensive research on final ES for decision support, including a five-year effort documenting the incorporation of final ES production and benefits into community-scale decision making [157–159]. The Community-Based Final Ecosystem Goods and Services Project incorporated five coordinated case studies in the Great Lakes, Puerto Rico, Pacific Northwest, Gulf of Mexico, and US Southern Plains [157]. Although work in each of the case studies encompasses multiple elements of the conceptual model (Figure 1), for brevity only a high-level overview of some of the conceptual model components for each case study is presented below. These coordinated case studies explored a number of elements common among the studies to inform the application of the conceptual model (Figure 1) to real-world issues, specifically focusing on understanding the challenges of operationalizing the framework, and developing lessons learned and elements of transferability for future case studies [157]. An important aspect of these case studies is the integration of multiple conceptual model elements to inform decision support.

### 4.1. Final ES Research in the Great Lakes

The Great Lakes case study aimed to expand existing processes (e.g., sediment remediation, water quality improvements, and aquatic habitat restoration) to remove BUIs with the inclusion of ES concepts and engagement of different suites of stakeholders [157]. The goal was to incorporate ES into decision making by providing information regarding how decisions affect ES within existing, agreed upon programmatic targets for remediation, restoration, and revitalization (referred to as the R2R2R framework) [119]. Williams et al. [160] provided a history of the St. Louis River Estuary addressing the role of community support in environmental remediation and community revitalization, concluding that community engagement empowers the community to take collective action to improve water quality, resulting in restoration of environmental benefits to waterfront communities, and provides the foundation for new, sustainable economic activity and development [161]. Angradi et al. [161] compiled environmental, social, economic, and governance indicators and metrics of revitalization from community revitalization and sustainability plans that can be used to plan for comparing alternative designs and to track restoration progress.

Williams et al. [6] identified forces that shape decisions, participation, and the inclusion of stakeholders and public values (i.e., ES) in the St. Louis River Estuary AOC indicating that the value of ES is context-dependent and changes according to an agency’s, organization’s, or individual’s relationship to an environmental resource. From a survey and interviews conducted with managers and advisory committee leaders, Holifield and Williams [162] suggested that recruiting and integrating participants and sustaining participation over the long term presents distinctive, ongoing challenges not fully recognized in existing processes of stakeholder participation. Focused on encompassing a suite of different approaches about scale and boundaries of water governance in environmental decision making, Holifield and Williams [162] developed an expanded conceptual model of stakeholder participation. Williams and Hoffman [119] integrated an HIA framework into the R2R2R framework, testing an HIA application in a habitat restoration project to identify and connect potentially impacted ES as a result of the project to health benefits endpoints such as swimmable waters or edible fish. Overall, these efforts provide tools and approaches to understand the relationships between US EPA programs, states, and communities, to facilitate communication and cooperation [6]. Importantly, this model functions as a “translator” approach, designed to identify, facilitate, and interpret distinct values among parties in a multi-entity governance effort, and is applicable to other environmental decision contexts, both within and outside the US.

### 4.2. Final ES Research in Puerto Rico

The Puerto Rico case study used tools and approaches to investigate impacts of alternative decisions on ES and their social and economic benefits [163], focusing on specific SDM elements: (1) clarify the decision context; (2) characterize goals and metrics; (3) develop information to link decision alternatives to changes in ES; (4) link decision alternatives to effects on human health and well-being; and (5) use an integrated, spatially explicit modeling framework to evaluate tradeoffs [159]. An important, transferable lesson focused on the value of engaging stakeholders early and often in the decision process [164].

The Guánica Bay, Puerto Rico, watershed has been a priority for research, assessment, and management since the 1970s, including an emphasis on addressing effects of land management decisions on coastal resources. A 2008 Watershed Management Plan characterizing a suite of proposed management actions to reduce sediment runoff and harmful effects in the coastal zone served as the initial decision context for the US EPA’s research [164]. Carriger et al. [165] used information from this plan, federal and common-wealth agencies, and nongovernmental organizations to apply qualitative decision-analysis structuring methods (focusing on identifying: overall objectives; fundamental objectives; and exploring means to achieve them) to gain insight into desired and undesired prospects. Conducting workshops with stakeholders, experts, and decision makers to explore past decisions, Bradley et al. [164] characterized the decision landscape to better understand multiple values and perceptions of citizens in different communities of the watershed, resulting in a more comprehensive decision landscape (i.e., beyond coral reef protection) and suite of decision alternatives.

Conceptual frameworks such as the Driver–Pressure–State–Impact–Response (DPSIR) framework provide a mechanism for planning and organizing information, identifying knowledge gaps or stakeholder concerns, identifying metrics or indicators for assessment, and providing the conceptual basis for mathematical models. Yee et al. [166] applied the DPSIR framework to two distinct case studies: (1) development of water quality criteria for protecting natural integrity of coral reefs; and (2) implementation of the 2008 Watershed Management Plan to protect coral reefs. In both cases, sketching an initial DPSIR aided in clarifying the decision context, including framing the decision and goals, illuminating the range of objectives and alternatives under consideration, visualizing potential tradeoffs, identifying key stakeholder groups who may have been impacted by decisions, and planning what was needed for analysis [166]. Bradley and Yee [167] extended the DPSIR framework by integrating human health and ecosystem health into a single framework, making it more broadly applicable to issues of sustainability. Applying a systems-thinking framework, such as DPSIR, within a structured decision-making approach better enables marine ecosystem managers to utilize scientific information toward more sustainable decision making [166].

One approach for characterizing where additional scientific research would best support improved decisions and resolve possible conflicts among stakeholders over preferred management actions using a normalized metric is the Expected Consensus Index of New Research [168]. This method was applied to coral reef protection and restoration in the Guánica Bay watershed, focusing on assessing and managing anthropogenic stressors, suggesting that new scientific research would be likely to bring people who initially disagreed to consensus, providing useful insights into social implications of a research program [168].

In a pair of studies, Refs. [45,163] examined integrated assessment approaches for connecting ES, human well-being, and the economic, social, and ecological elements of sustainability decision making. Their approach involves mapping and quantifying indicators of ES and human well-being at a neighborhood-scale to examine whether ES information explains variability in elements of human well-being. These integrated assessment approaches can be used for estimating potential benefits and tradeoffs in terms that are meaningful to people living in a community.

### 4.3. Final ES Research in the Pacific Northwest

A case-study research in the Pacific Northwest, focusing on several different watersheds with unique stakeholders and watershed impairment issues, used a common decision-support approach focusing on transferable modeling tools and co-development of alternative model-based decision scenarios among stakeholders [157].

One area of emphasis focused on understanding forest management practices that most effectively restore salmonid populations while providing clean drinking water, forestry jobs, and cultural benefits for local communities and Tribes. In the Willamette River Basin (Oregon), Bolte et al. [169] presented ES research using the Envision tool, a multi-agent model used to generate alternative future scenarios that show how possible decision choices interact and collectively impact a landscape’s capacity to supply ES of interest. McKane et al. [41] used the Envision tool and the VELMA model to assess ES relevant to rural and downstream urban communities resulting from alternative forest management and climate scenarios in the Willamette River Basin and Puget Sound (Washington).

Additional research in the Tillamook Bay (Oregon) watershed focused on identifying management practices that reduced nutrients, sediments, and fecal matter flowing down to the estuary, and how to prioritize these practices to protect a range of objectives related to human health (e.g., clean drinking water) and sustainable local economies (e.g., shellfish production) [157]. Estuarine ES–related research included intermediate ES research on habitats (e.g., overall habitat suitability) [170,171] and environmental stressors such as macroalgal blooms [172] and fecal coliform levels [173] supporting environmental decision making related to shellfish final ES.

### 4.4. Final ES Research in the Gulf of Mexico

A case-study research in the northern Gulf of Mexico has recently focused on Mobile Bay (Alabama) and desired sustainability, resilience, and restoration benefits for the Mobile Bay National Estuary Program [157]. The goals of this case study focused on how potential restoration activities (e.g., improving stream water quality and shoreline health) could be examined in the context of ES provisioning and how services provided to people (a key measure of success) might be impacted by changes in land use. Targeted research focused on: (1) applying SDM approaches; (2) working with stakeholders to identify their fundamental objectives; (3) characterizing measures of community well-being; (4) evaluating potential management actions related to their fundamental objectives; and (5) linking these to the production of ES that directly benefit the community [157]. Evaluating elements of transferability of quantitative tools and strategies to examine similarities and differences among communities is an additional area of focus.

### 4.5. Final ES Research in the Southern Plains

The Southern Plains case study focused on issues around community water supply, specifically the multifaceted, multi-stakeholder planning processes needed to develop community sustainability and resiliency plans and those related ES such as flood control, recreational activities, irrigation, and wildlife habitat [157]. One of the US EPA’s tools in development is the web-based application Decision Analysis for a Sustainable Environment, Economy, and Society (DASEES) built around a five-step, iterative decision process focused on: (1) understanding the decision context; (2) defining objectives; (3) developing options; (4) evaluating options; and (5) taking action. In the Southern Plains, the DASEES application was used to assist multiple communities in resiliency planning focused on those ES that require shared cooperation (e.g., water supply and flood protection) in addressing common issues in water resource management and expected population increases. As an aside, another example of DASEES application is presented by Dyson et al. [174].

## 5. Conclusions

An explosion in ES science started in 1997 with two seminal publications [1,2], triggering an extensive amount of research [3]. Research by the US EPA has incorporated multiple dimensions of ES science (Figure 1).

A suite of challenges and opportunities include: developing an operational understanding of the final ES conceptual model; matching EPFs and final ES metrics to the problem at hand; developing a “toolbox” for ES tools and approaches (e.g., a workbook to choose among standardized final ES metrics); improving stakeholder involvement in decision making; and continuing the need to integrate multiple issues into the decision process. Lessons from research to advance each of the final ES elements in Figure 1 can be useful for identifying gaps and future science needs. Additionally, recent efforts to synthesize and evaluate the US EPA’s overall portfolio has highlighted understanding important elements of Transferability, Uncertainty, Scalability, and Communication in a strategic manner (referred to as TUSC). DeWitt et al. [8] summarized the final ES science that will be needed for advancing ecosystem-based management, identifying the following needs:

- Greater awareness within the [ecosystem-based management] community of practice, including developing case-study applications, of the usefulness of [final ES] and the availability of tools useful for identifying, prioritizing, and quantifying them.
- A standardized list of metrics or indicators for each [final ES], based on the attributes of ecosystem types that each beneficiary class uses, enjoys, or appreciates. Site-specific metrics or indicators could then be developed from those generic attributes.
- Integration of the [final ES] tools (e.g., NESCS Plus, FEGS Scoping Tool, Rapid Benefits Indicators, EcoService Models Library) to facilitate identification of priority [final ES], relevant metrics and indicators for [final ES] endpoints and benefits, and models for estimating responses of those [final ES] to environmental change or stressors ([8] p. 129).

In the context of transferability, current and future US EPA research focuses on translating and applying final ES concepts for different decision contexts. These include applications to ecological risk assessments, National Environmental Policy Act processes, cleanup of contaminated sites (e.g., Superfund, AOC, and R2R2R contexts), as well as different fields such as ecosystem condition assessment, ecosystem restoration, and informing disturbance recovery. In an example describing where novel, final ES research is developing, Yee et al. [175] described how to translate final ES science into informing environmental conditions assessments using an ES gradient approach that provides meaningful measures of ecosystem change; how changes in ecosystem condition affect ES; and how to communicate the resulting tradeoffs and benefits to decision makers and stakeholders. Overall, the broad suite of research described in this paper has identified the need for consistent application and integration of core final ES elements into a cohesive approach [8], resulting in the integration of science and policy to improve environmental, community, and sustainability decision outcomes.

## Figures and Tables

**Table 1.** Structured process for proposing final ES metrics. Source: modified from [101].

Step	Description
Step 1: Ecosystem Delineation	Explain how to define the boundaries of the ecosystem(s) of interest for practical purposes.
Step 2: Beneficiary Specification	Describe the beneficiaries to be considered for each ecosystem.
Step 2: Attribute Specification	Identify the biophysical components of nature that link with the ecosystem service and beneficiary’s interests.
Step 4: Metric Specification	Describe the units of the attribute and discuss the difference between available and ideal metrics.
Step 5: Data Availability	Consider the availability of appropriately selected data for the proposed metric.

## Data Availability

All results and secondary data mentioned in this Critical Review are publicly available and described in the References section.
